# Encapsulation of biocides by cyclodextrins: toward synergistic effects against pathogens

**DOI:** 10.3762/bjoc.10.273

**Published:** 2014-11-07

**Authors:** Véronique Nardello-Rataj, Loïc Leclercq

**Affiliations:** 1 Université de Lille, Sciences et Technologies, EA 4478, Chimie Moléculaire et Formulation, F-59655 Villeneuve d’Ascq Cedex, France

**Keywords:** cyclodextrin, biocide, encapsulation, host–guest chemistry, pathogen, textile

## Abstract

Host–guest chemistry is useful for the construction of nanosized objects. Some of the widely used hosts are probably the cyclodextrins (CDs). CDs can form water-soluble complexes with numerous hydrophobic compounds. They have been widespread used in medicine, drug delivery and are of interest for the biocides encapsulation. Indeed, this enables the development of more or less complex systems that release antimicrobial agents with time. In this paper, the general features of CDs and their applications in the field of biocides have been reviewed. As the key point is the formation of biocide–CD inclusion complexes, this review deals with this in depth and the advantages of biocide encapsulation are highlighted throughout several examples from the literature. Finally, some future directions of investigation have been proposed. We hope that scientists studying biocide applications receive inspiration from this review to exploit the opportunities offered by CDs in their respective research areas.

## Introduction

Since the first reference to cyclodextrins (CDs) in 1891 by Villiers, CD has been of great interest to researchers [1]. Cyclodextrins are composed of several α-D-glucopyranose units linked 1→4 and arranged in a conical shape [2]. There are three principal types named α-, β-, and γ-CDs, which are composed of 6, 7, and 8 glucose units respectively. The primary hydroxyl groups are orientated to the narrow edge of the cone whereas the secondary hydroxyl groups at the wider edge. These hydroxyl functions allow an easy solubilization of CDs in water. In contrast, the cavity has a hydrophobic character due to carbons and ethereal oxygen atoms and allows the formation of inclusion complexes with hydrophobic molecules [3–5]. This property is useful to solubilize and to stabilize highly hydrophobic molecules in aqueous environment. However, inclusion complexes in the solid state can also be obtained [6]. Since the water-solubility of native CDs ranges from 18 to 232 g/L, a variety of modified CDs has been developed and most widely employed to improve the formation of inclusion complexes and their solubility [7]. Indeed, the hydroxyl groups allow the introduction of various functional groups [8]. As example, some native and chemically modified CDs are presented in Table 1.

**Table 1:** Structures and acronyms of some natives and modified cyclodextrins reported in this review.^a^

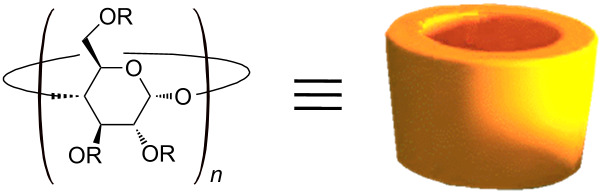		

*n*	Abbreviation	Residue

*n* = 6	α-CD	R = H
*n* = 7	β-CD	
*n* = 8	γ-CD	
*n* = 7	HP-β-CD	R = H or –CH_2_CH(OH)CH_3_
*n* = 8	HP-γ-CD	
*n* = 7	S-β-CD	R = H or –SO_3_Na
*n* = 7	MCT-β-CD	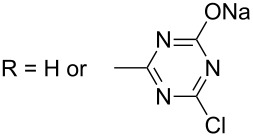
*n* = 7	SBE-β-CD	R = H or –(CH_2_)_4_SO_3_Na
*n* = 7	CM-β-CD	R = H or –CH_2_CO_2_Na

^a^HP: 2-hydroxypropyl; S: sulfo; MCT: monochlorotriazinyl, SBE: sulfobutyl ether, CM: carboxymethyl.

The main reasons why CDs are popular for inclusion of various molecules are the following: i) they are produced from a renewable natural material (i.e., starch) applying environmental-friendly technologies (i.e., enzymatic conversion), ii) they are relatively cheap and are produced in amounts of thousands of tons per year, iii) their chemical modification is relatively easy, iv) they are nontoxic in consumable concentrations (i.e., CDs are biocompatible), and v) they are biodegradable and do not pollute the environment. Their main fields of application are: agriculture, food industry, biotechnology, pharmaceutical industry, chemical and biological analysis, chemical synthesis, catalysis, materials, cosmetic industry, environmental protection technologies and textile industry [9–13].

Although the application of CDs to the biocide field is not really new, there is still room for the development of new systems with advanced properties. The objective of this contribution is to focus on the use of natural and chemically modified CDs to improve the performances of biocides.

## Review

### Biocides versus pathogen agents: the context

#### i) Definitions and markets

Pathogen is used to mean an infectious agent (e.g., virus, bacteria, prion, fungus or protozoan). The host may be an animal, a plant, a fungus or even another microorganism. To prevent and to treat pathogen infections, biocides are commonly used. Biocidal products are defined as “*active substances and preparations containing one or more active substances, put up in the form in which they are supplied to the user, intended to destroy, render harmless, prevent the action of, or otherwise exert a controlling effect on any harmful organism by chemical or biological means*” (Directive 98/8/EC of the European Parliament and Council of the 16 February 1998, Article 2.1.a). Due to their use in medicine, agriculture and industry, the market of biocides is still in expansion [14].

#### ii) Common biocides and modes of action

As the number of biocides in use is large, some active substances as well as their mode of action are listed in Table 2 with a classification based on active chemical groups.

**Table 2:** List of active molecules used in biocidal formulations (in bold: active biocides mentioned in this review) and their mode of action classified on the basis of active chemical groups.^a^

Active chemical groups	Active molecules	General mode of action

Alcohols	Ethanol Benzyl alcohol	Inhibition of DNA and RNA synthesis
Aldehydes	Glutaraldehyde Formaldehyde	Alkylating agents
Amphiphiles	**Didecyldimethyammonium chloride** **Benzalkonium chloride**	Membrane destabilizer, cytoplasmic protein aggregation
Azole derivatives	**Enilconazole** **Miconazole** **Tebuconazole**	Inhibition of the ergosterol synthesis, interaction with DNA
Biguanides	Chlorhexidine	Inhibition of membrane-bound adenosine triphosphatase (ATPase)
Carbamates	**Bendiocarb** **Carbendazim** Carbofuran	Inactivation of the acetylcholinesterase
Halogens	Chlorine Iodine	Oxidizing agents
Heavy-metal derivates	**Copper** **Silver**	Interaction with thiol residues
Neonicotinoids	**Imidacloprid** Sulfoxaflor	Binding to nicotinic acetylcholine receptors
Organic acids	Lactic acid ***p*-Hydroxybenzoic acid ester** **Butylparaben**	Dissipation of proton motive force and inhibition of uptake amino acids
Organophosphates	**Chloramidophos** **DCPE**	Inactivation of the acetylcholinesterase
Phenol, Bisphenol and derivatives	*m*-Cresol **Pentachlorophenol** **Triclosan**	Inhibition of uptake amino acids, modification of membrane potential
Pyrethroids	Allethrin **Cypermethrin**	Modification of membrane potential
Quinolones	**Ciprofloxacin** Levofloxacin	Inhibition of the topoisomerase
Terpenes	Limonene ***trans*-cinnamaldehyde** **Eugenol**	Unknown membrane interaction

^a^Note that the list is not exhaustive. The reader can consult reference [15] for more details.

As depicted in Table 2, the mechanism of action depends on the molecular structure of the biocides. Moreover, the concentration used has also a considerable influence on the mechanism. As examples at relatively low concentrations, the amphiphilic agents (surfactants) are membrane permeabilisers whereas at high concentrations, the penetration of these compounds in the cytoplasmic leads to protein aggregation [15]. It is worth mentioning that the additives may also have an effect on the biocidal activity (pH, surfactants, antioxidants, chelating agents, etc*.*) [16–17]. Moreover, some formulations contain mixtures of biocides in order to obtain additive or synergistic effects [18–21].

#### iii) Problems associated with the use of biocides

**Scheme 1:**
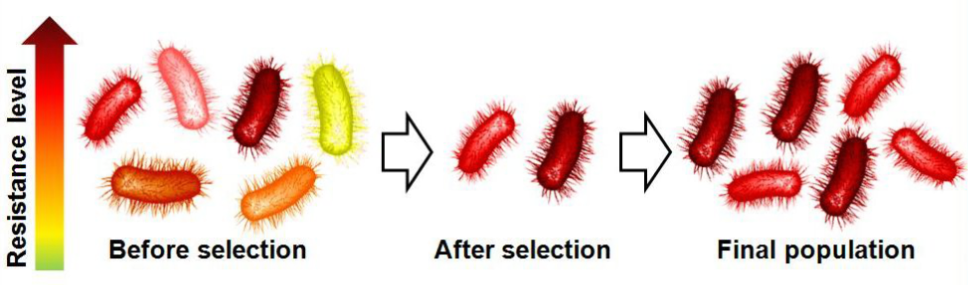
Principle of resistance mechanisms through selection of the most resistant micro-organism.

Whatever the biocidal mechanism, the pathogen agents are killed or inhibited. However, pathogens might adapt to new environmental conditions: repeated exposures to disinfectants increase the selective pressure and allow the emergence of resistant pathogens (Scheme 1) [16]. The emergence of resistance strains to biocides which, in some cases, can contribute to resistance to antibiotics becomes a major problem. Microorganisms are considered resistant to antibiotics or biocides when a strain is not killed or inhibited by: i) the biocidal concentration typically used in practice, ii) a concentration at which the majority of strains of that microorganism is affected, and iii) a concentration acting upon the majority of cells in that culture [22].

Moreover, biocides are chemical agents that are intrinsically usually toxic for the end user, but also for the environment [23]. The toxicity of some biocides has been particularly well described. As examples glutaraldehyde and amphiphilic ammonium have been associated with dermatitis [24–25]. Toxicity, hypersensitivity and irritation have also been reported with other biocides such as formaldehyde [26], chlorhexidine [27] and iodine [28]. In the case of pesticides, their impact on the environment is not harmless (e.g., some herbicides have an influence on bees) [29]. To improve their bioactivity as well as their stability on storage, their physicochemical characteristics, biocides can be easily encapsulated by CDs. In this review, the advantages of using CDs in the presence of biocides will be discussed and illustrated throughout several examples from the literature. It is noteworthy that the review is divided in three parts devoted to three biocide families: organic, amphiphilic and heavy-metal biocides. The final section is devoted to the intrinsic biocidal activity of CDs to obtain synergism.

### Organic biocides

Benefits that can be achieved when CDs are combined with organic biocides are numerous. Indeed, the complexation of the biocide by the CD improves: i) the physicochemical properties of the biocides (e.g., increased aqueous solubility and wettability, reduced vapor pressure, etc.), ii) the controlled release and bioavailability, iii) the shelf-life, iv) the storage conditions and the environmental toxicity, and v) the biocidal property of textiles which is one of the main fields of application of CD–biocide complexes and which will be developed in this review as an example. In this section, all these key advantages are illustrated by some references taken from the literature. However, it is worth mentioning that the separation of the advantages of CDs applications in given sections is purely fictive. Indeed, the combination of advantages is often reported.

#### i) Improvement of water-solubility of organic biocides

Commonly, biocides are used in aqueous solution. However, the most highly effective organic biocides have an extremely low water-solubility. Therefore, additives (e.g., detergents or organic co-solvents) are required. However, these additives and the resulting complex formulations often lead to undesired interactions and/or precipitations. Moreover, their impact on the environment enhances the toxicity of the formulation. In the literature, the formation of biocide–CD inclusion complexes is presented as a very attractive tool to modify the physicochemical properties of an organic biocide. Indeed, upon complexation, the lipophilicity can be disguised and the aqueous solubility can thus be enhanced.

**Figure 1:**
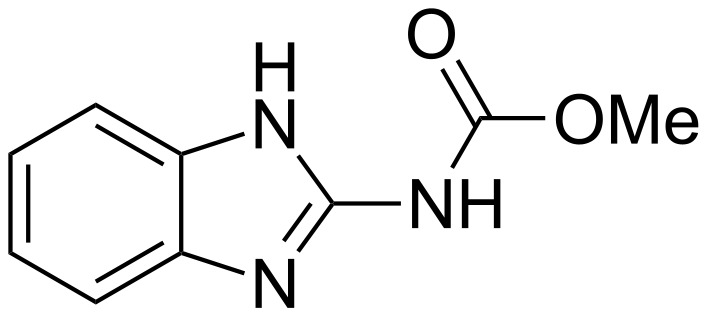
Chemical structure of carbendazim.

One of the most useful and widely applied analytical approaches to determine the CD effect on the solubility is the “phase-solubility” method described by Higuchi and Connors in 1965 [30] and applied to various guest [31–35]. As results, the encapsulation of numerous organic biocides has been reported in the literature (e.g., thiabendazole, fuberidazole, 3-chloro-*p*-toluidine, etc.) [36–37]. It is noteworthy that this list is by far not exhaustive and numerous other examples of biocide–CD inclusion complexes are found in the literature. In the present section, for sake of clarity, only one typical example of CD encapsulation is reported. However, upon CD complexation, all organic biocides developed in the following sections also present an enhancement of their water-solubility in addition to the other benefits highlighted. The methyl *N*-(1*H*-benzimidazol-2-yl)carbamate (named carbendazim, see Figure 1) was used to illustrate the effect of CDs on its aqueous solubility.

Carbendazim is a widely used systemic fungicide against different fungi affecting plants [38–39]. Its mechanism is linked to its antimitotic properties that inhibit the bacterial growth [40]. Due to its poor water-solubility, its application becomes a major constraining factor.

In 1996, Schmidt et al. have mentioned in a patent that the water-solubility of carbendazim can be improved by formation of a complex with CDs [41]. However, the first academic study dealing with the increase of the water-solubility of carbendazim upon its complexation with native α- and β-CDs has only been reported in 2002 by Lezcano et al. [36]. Phase solubility and fluorescence emission spectra of the carbendazim in the presence of different CD concentrations have been applied to characterize the inclusion complexes. In each case, 1:1 inclusion complexes were observed between carbendazim and CD. However, the binding constants are very weak (14.2 and 22.7 M^−1^ for the α- and β-CDs, respectively). These results suggest that the benzimidazole nucleus can be more easily complexed by β-CD (cavity diameter = 6.0–6.5 Å) than by α-CD (cavity size diameter = 4.7–5.3 Å). With the latter, the authors have suggested that the methyl carbamate residue is the binding site. Despite the weak binding constants, the carbendazim water-solubility is increased but the effect remains low (1.9-fold compared to free carbendazim in the presence of 15 mM of β-CD).

In 2012, Ge et al. proposed to use the HP-β-CD to enhance the carbendazim solubility [42]. From the method of continuous variations (Job’s method), it was found a 1:1 stoichiometry for the carbendazim–HP-β-CD inclusion complex. The complex was fully characterized by phase-solubility diagram, fluorescence, ^1^H and ROESY NMR and FTIR experiments. The binding constant of the complex was estimated at 61.07 M^−1^. As expected, this inclusion complex was found to notably increase the aqueous solubility of carbendazim compared to its free solubility (4.2-fold in the presence of 50 mM of HP-β-CD). NMR experiments indicated that the benzene ring of carbendazim was complexed. In addition, the authors have reported that the complexation of carbendazim with HP-β-CD significantly improves its bioavailability and, therefore, resulting in about a two-fold increase of the fungicidal activity against *Trichioderma viride* (fungus) compared to carbendazim alone. As a consequence, complexed carbendazim allows a decrease of the amount of organic solvents used to reach the same activity.

#### ii) Antagonistic effect

The modification of the physicochemical parameters upon complexation can be seen as an antagonistic effect when the combined effect results in a lower biocidal activity than the activity of the biocide alone. In 1993, Lehner et al. reported a very interesting study on the antagonistic effect of the complexation between HP-β-CD and a series of *p*-hydroxybenzoic acid esters against *Candida albicans* [43]. The authors investigated the complexation in aqueous solution by solubility isotherms and in the solid state by thermal analysis (DSC) and X-ray diffractometry. The variation of the alkyl chain structure of the *p*-hydroxybenzoic acid esters revealed that the stability of the inclusion complexes was deeply modified (see binding constants in Table 3).

**Table 3:** Binding constants of HP-β-CD inclusion complexes with various *p*-hydroxybenzoic acid esters and minimal inhibition concentrations (MIC) for free and complexed *p*-hydroxybenzoic acid esters against *C. albicans*.^a^

*p*-hydroxybenzoic acid esters	*K*_1:1_	MIC (µg/mL) of free esters	MIC (µg/mL) of 1:1 complex

methyl	969	750.2	3 300.7
ethyl	948	519.7	1 900
propyl	1 548	200.0	579.9
butyl	3 352	70.0	139.9
amyl	4 760	55.0	130.0
benzyl	6 039	65.0	120.1

^a^Taken from [43].

As depicted in Table 3, the antifungal effect of *p*-hydroxybenzoic acid esters against *C. albicans* is directly linked to their hydrophobicity: the minimal inhibition concentrations were directly correlated to the nature of the residue. Table 3 also highlights that the antimicrobial activity of the preservatives is reduced in the presence of HP-β-CD. Based on quantitative structure activity relationship analyses, this effect can be described by physicochemical parameters such as hydrophobicity or complex stability constants. Indeed, upon complexation, the water-solubility of the preservatives is increased and the concentration of free *p*-hydroxybenzoic acid esters decreases with the binding constant increase. These results suggest that the degree of inactivation is completely dependent on the encapsulated fraction of the *p*-hydroxybenzoic acid ester. It is worth mentioning that the “deactivation” of parabens by CDs is less pronounced with α- than β-CD [44]. Indeed, interactions between a series of *p*-hydroxybenzoic acid esters (from methyl to dodecyl residues) with α-CD than with β-CD have been investigated by Uekama and coworkers [44]. In the presence of both types of CD, the antimicrobial activity against *C. albicans* was decreased but the extent of the “inactivation” is quite different: the MIC of free methylparaben was estimated at 750 µg/mL but its value was increased up to 1 497 and 1 915 µg/mL for α- and β-CD (10^−2^ M), respectively. Therefore, the biocidal activity depends on the magnitude of the stability constants (218 and 870 M^−1^, respectively for α- and β-CD) as they play a role on the concentration of free alkylparaben in aqueous solution.

In 1994, the same authors extended their study to other biocides (e.g., bronopol, thimerosal, chlorhexidine diacetate, *p*-chloro-*m*-cresol, phenylmercury acetate, propyl *p*-hydroxybenzoate, benzyl alcohol, 2-phenoxyethanol) [45]. The authors established a clear relationship between the binding constants and the antimicrobial activity of the preservatives on various strains: *Staphylococcus aureus*, *Pseudomonas aeruginosa*, *Escherichia coli* and *C. albicans*. Indeed, highly water-soluble substances (e.g., thimerosal and bronopol) showed low inactivation whereas lipophilic substances (i.e., phenolic residues) presented strong inactivation in the presence of HP-β-CD. This behavior is due to the sequestration of the more hydrophobic biocides by CDs. Therefore, the appropriate biocide and CD must be selected to maintain an acceptable biocidal activity. Beyond these considerations, many benefits can be obtained by the use of CDs (see below).

#### iii) Controlled release and bioavailability improvement

**Scheme 2:**
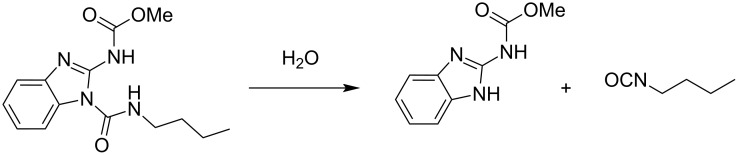
Chemical structure of benomyl and its decomposition in aqueous solution.

As mentioned above, the formation of inclusion complexes can be useful to enhance the bioavailability of biocides. As an example, Szejtli et al. reported that the biocidal activity of methyl [1-(butylcarbamoyl)benzimidazol-2-yl]carbamate (named benomyl) can be improved in the presence of β-CD (Scheme 2) [46]. Indeed, the same fungicidal efficiency can thus be achieved with a lower active ingredient dose. The authors reported that when the inclusion complex is used directly with solid culture medium, a fungistatic activity against *Alternaria tenuis*, *Rhizoctonia solani* and *Sclerotinia sclerotiorum* is obtained between 0.1 and 20 µg/mL. The fungistatic effects manifest in the modification of morphologies, structures and colors of colonies. For higher dosage, a clear decrease in the diameter of the colonies is also reported. In contrast, the use of the biocide alone has no impact on the culture medium in the same range of concentrations. Moreover, this carbamate, which is a systemic fungicide, has a poor water-solubility and presents some decomposition to carbendazim which also has a fungicidal effect but which remains little soluble in water (see above and Scheme 1) [47]. Hence, the authors claimed that the synergistic effect is due to a better dissolution of the active ingredient. Indeed, as the complex is characterized by a dynamical equilibrium (≈10^−8^ s^−1^), the active ingredient incorporated into the CD acts in the same way from the point of view of absorption as the free dissolved active ingredient. Thus the concentration of dissolved carbendazim by CDs suitable for direct absorption is significantly increased. In other words, the rate of absorption is accelerated because of bioavailability improvement.

**Figure 2:**
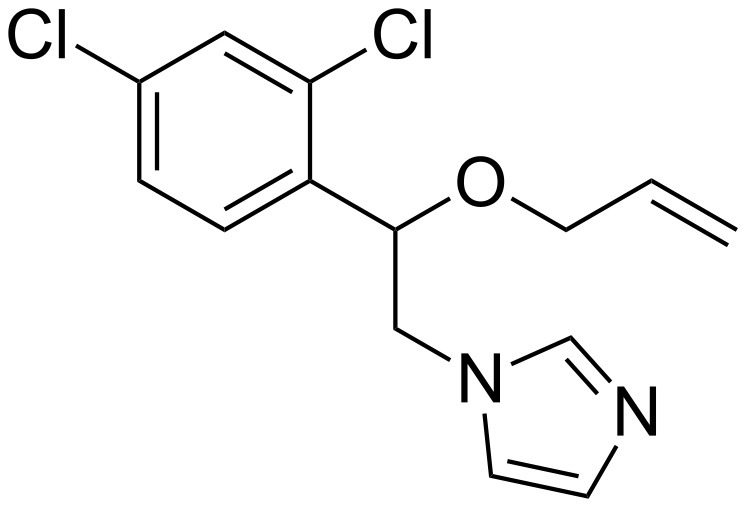
Chemical structure of enilconazole.

In 2002, Schirra et al. reported on the inclusion of 1-[2-(allyloxy)-2-(2,4-dichlorophenyl)ethyl]-1*H*-imidazole (named enilconazole, see Figure 2) in the β-CD and the antifungal activity of the 1:1 inclusion complex against *Penicillium digitatum* and *P. italicum* [48]. The freshly prepared enilconazole–β-CD complex was as effective as free imazalil. However, after 4 days, the enilconazole–β-CD complex was more effective against the considered microorganisms. The authors proposed that enilconazole was gradually released from the complex, thereby increasing its bioavailability.

#### iv) Improvement of shelf-life

**Figure 3:**
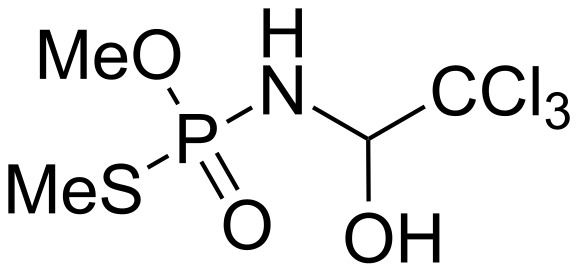
Chemical structure of chloramidophos.

Upon complexation, the shelf-life of biocides can be improved (e.g., stability to heat, light, oxygen and even stability to microorganisms). In 2007, Zhang et al. solved the problem of unstability of chloramine phosphate (chloramidophos, see Figure 3) as a pesticide by the formation of 1:1 inclusion complexes with β-CD [49].

The formation of inclusion complex was proved by DSC, Fourier transform infrared and X-ray diffraction [50]. The binding constant was estimated at 203 M^−1^ from UV–vis spectrophotometry. The degradation rate of chloramidophos in 14 days incubation was slowed down by a factor of 3.6 when complexed with β-CD. The authors reported that the biocidal mechanism is not affected by the organophosphorus complexation. Indeed, the formation of chloramidophos–β-CD inclusion complexes greatly improves the thermal stability of the biocide with no negative influence on its activity and toxicity.

In 2013, Hill et al. reported that β-CD can be used to encapsulate various essential oil constituents (e.g., *trans*-cinnamaldehyde, eugenol, etc.) for antimicrobial delivery applications [51]. The inclusion complexes were prepared by the freeze-drying method and characterized by transmission electronic microscopy, oxidative DSC and phase solubility. The encapsulation efficiency was very high for all the essential oils. The phase solubility method was typical of a 1:1 molecular ratio for all complexes and the binding constant was estimated at 28.5 and 174.5 M^−1^ for *trans*-cinnamaldehyde and eugenol, respectively. The analysis of the antimicrobial activity of complexes against *Salmonella enterica* serovar Typhimurium LT2 and *Listeria innocua* strains revealed an inhibition of the bacterial growth. The inclusion complexes were able to inhibit the two strains at lower active compound concentrations than free oils because of their better water-solubility (see above). Moreover, the encapsulation of oils masks the flavor but protects also against oxidation or heat damage. Indeed, the oxidative DSC allowed the comparison of thermal oxidation stability of free oils and encapsulated ones. The exothermic peaks at approximately 265 and 260 °C, which result from the hydrolysis and oxidation of *trans*-cinnamaldehyde and eugenol, were not detected for the corresponding inclusion complexes. Therefore, the essential oils were protected by the β-CD encapsulation. As a consequence, the essential oils remain effective as antimicrobial agents for longer time periods and under a wide variety of environmental conditions.

**Scheme 3:**
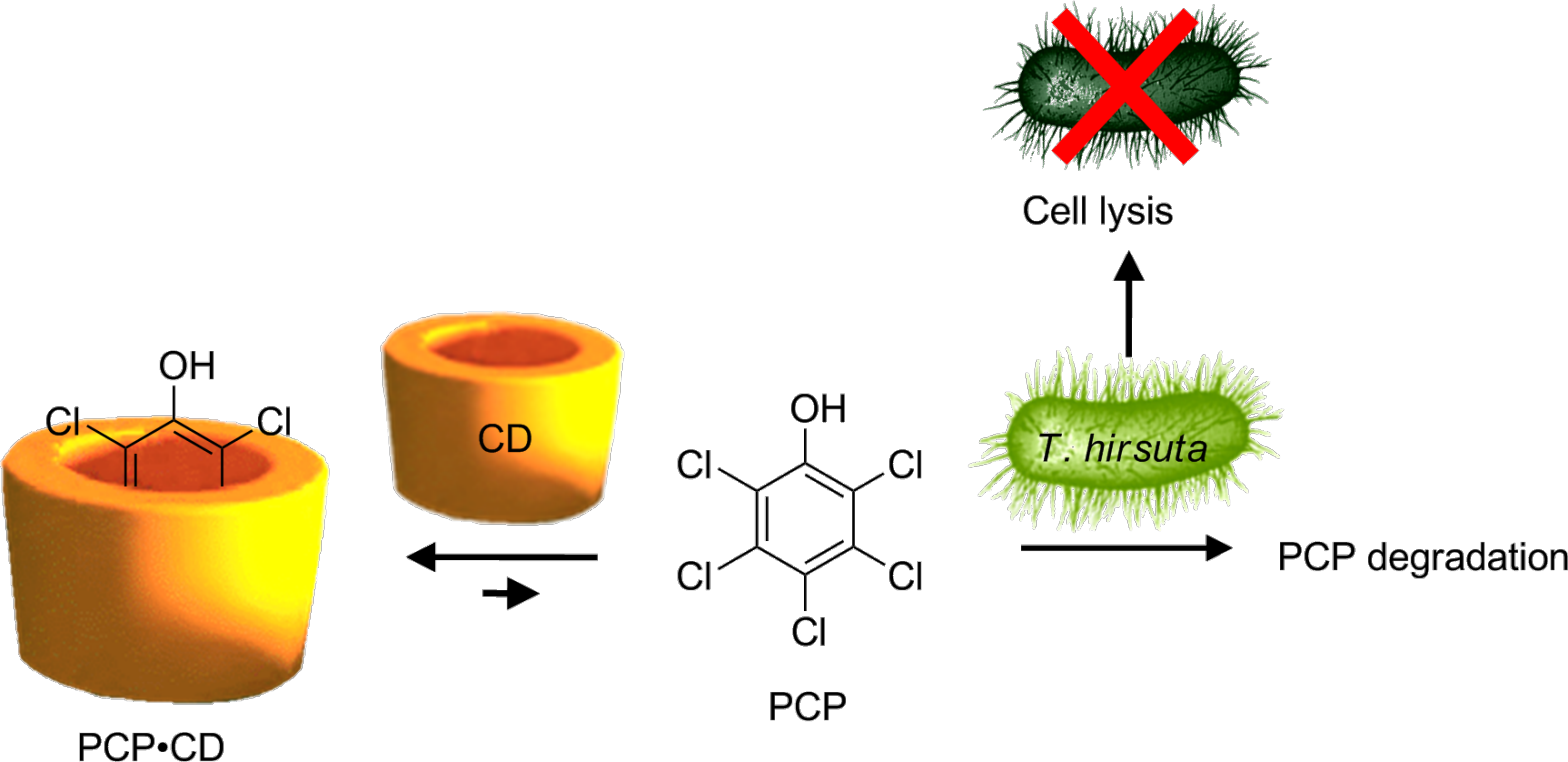
The complex problem of pentachlorophenol (PCP) degradation.

On the other hand, the biodegradation of hydrocarbons is influenced by their bioavailability [52]. Indeed, hydrocarbons are very poorly soluble in water. Therefore, their metabolization by microorganisms is very difficult. To overcome this drawback, surfactants are used to improve their aqueous solubility and their bioavailability in order to accelerate the degradation process. As mentioned above, CDs are of interest in these microbial processes because they form soluble complexes with numerous hydrophobic compounds but do not exhibit toxicity comparable with that of many chemical surfactants [53–54]. Since 1995, it is known that the degradation rate of phenanthrene by microorganisms is considerably accelerated in the presence of HP-β-CD [55–57]. However, the mechanism of biodegradation is not so simple. As an example, *Trametes hirsuta* (white-rot fungus) is used to decontaminate various substrates. One of them, the pentachlorophenol, is completely degraded by the microorganism via mineralization. However, as pentachlorophenol also acts as a biocide, the fungal growth is inhibited and thereby, its own degradation as well. In 2006, to overcome this drawback, Boyle reported that addition of γ-CD can reduce the intrinsic toxicity of pentachlorophenol, allowing thus the fungi to grow in the presence of relatively high pentachlorophenol concentrations [57]. Unfortunately, the mineralization was inhibited by this CD because of the formation of a too stable complex. Indeed, the complexed pentachlorophenol was not accessible to the degradative system of microorganisms. To overcome this drawback, the author proposed to use the CD at higher pH values. Under such conditions, the complex stability decreased as well as the pentachlorophenol toxicity. This example highlights the complexity of the introduction of CDs in a system based on multiple equilibria (Scheme 3).

#### v) Reduction of the environmental toxicity

**Figure 4:**
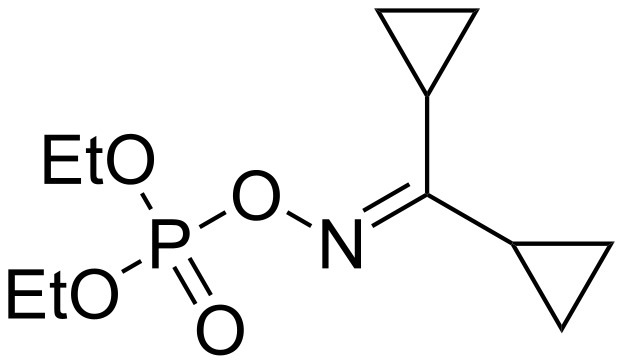
Chemical structure of DCPE.

The CDs can be used to minimize environmental pollution. As an example, in 1994, Loukas et al. reported the effect of γ-CD on dicyclopropylmethanone *O*-(diethoxyphosphoryl)oxime (named DCPE), a very active insecticide (Figure 4) [58].

The complexation of DCPE by the CD in aqueous solution was proved by ^1^H NMR. The stoichiometry of the DCPE–γ-CD complex was found to be 1:1 and the association constant was estimated at 1 488 M^−1^ at 35 °C. In the presence of 2.8 × 10^−3^ M of γ-CD, the stability of DCPE was enhanced by 2-fold. Moreover, the initial liquid biocide was transformed to a stabilized powder upon complexation. As CDs give a hydrophilic character to the biocide, the dermal absorption is reduced and the toxicity is decreased. As example, the LD_50_, determined on Wistar rats, is increased up to 6.5-fold between DCPE and its inclusion complex. It is thus shown that the CD complexation allows an easy storage, a decrease of the environment toxicity without a significant decrease of the anticholinergic activity.

**Figure 5:**
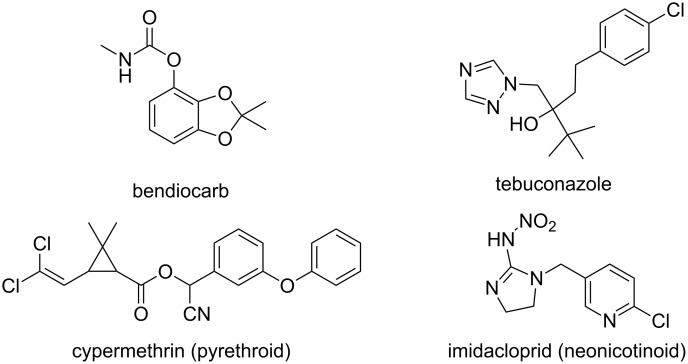
Chemical structures of some biocides used in [59].

Carbamate insecticides are less damaging for the environment, more biodegradable and less toxic for humans. However, insects become resistant to these biocides. In contrast, nicotinoid and pyrethroid do not have any disadvantage with respect to resistance. Since encapsulation of these pesticides can be easily performed with CDs, the stability of β-CD inclusion complexes of various biocides (bendiocarb, neonicotinoid and pyrethroid families and the tebuconazole; see Figure 5) under various storage conditions was studied by Alonso et al. [59]. As expected, the improvement of the water-solubility due to complexation increases the bioavailability of the biocides which are gradually released from the complex. Thanks to the micrometric size of the complex, the product is able to treat a larger surface and is less detectable for insects. In other words, the toxicological profile of the biocides is thus neutralized for the environment. Finally, the authors also mentioned that the solidification of liquid biocides is very useful for a better stability during storage. Indeed, thanks to the CD protection, the biocide degradation on storage is reduced and the difficulty arising in handling liquid biocides at ambient temperature is overcome. Finally, it is noteworthy that the release of included biocides from solid complexes is primarily due to the presence of water (i.e., wetting, dissolution or dilution in aqueous solution).

#### vi) Biocidal textiles

In the last decades, the application of antimicrobial agents on textiles for health care and hygiene applications received a lot of attention [60–62]. For sake of clarity, only some typical examples of CDs/biocide inclusion on textile are reported.

**Figure 6:**
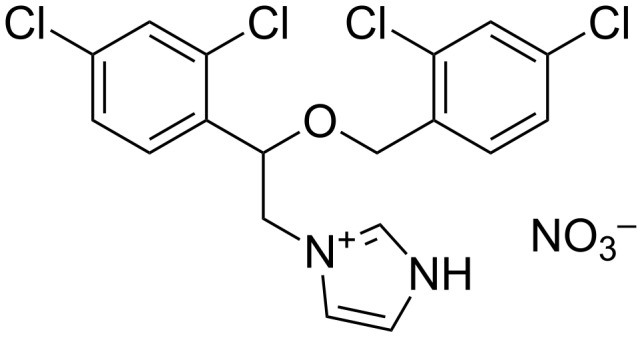
Chemical structure of miconazole nitrate.

In 2008, Wang et al. incorporated the miconazole nitrate (Figure 6) into the cavity of MCT-β-CD covalently bound onto cloth fibers. The miconazole salts are antifungal agents with additional antibacterial and antiparasitic actions [63]. The optimal conditions were obtained at 150–160 °C for 5–8 min, with MCT-β-CD 60–100 g/L, Na_2_CO_3_ 50–60 g/L leading to a grafting yield of about 5%.

UV spectrophotometry on modified and unmodified fabrics revealed that the miconazole nitrate entrapped in the functionalized textile with MCT-β-CD was higher compared to the unmodified one (up to 8.2-fold). As expected, the antifungal activity against *C. albicans* was enhanced for the MCT-β-CD grafted textile impregnated with miconazole nitrate. The finished fabric retains its antibacterial property even after ten washes unlike the unmodified textile. It is notable that the triazinyl group in MCT-β-CD itself is biocide providing some antimicrobial effects. Therefore, the combination of this CD and biocides is very promising.

**Figure 7:**
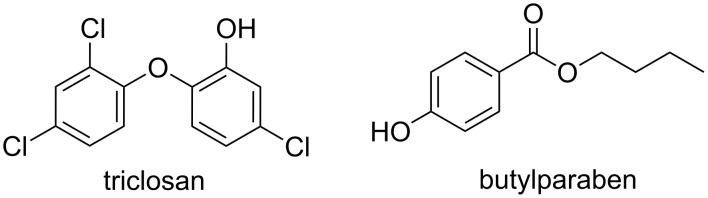
Chemical structures of triclosan and butylparaben.

In 2009, butylparaben and triclosan were immobilized into a cationic-β-CD polymer (CP-β-CD, see Figure 7) [64].

The cationic polymer (CP) was synthesized by one-step condensation of β-CD, epichlorohydrin and choline chloride at a fixed molar ratio of 1/5/2. At a CP-β-CD concentration of 6.0% (w/v), the water-solubility of biocides incorporated into the CP-β-CD cavities were significantly improved to 2.80 and 1.64 g/L for butylparaben and triclosan, respectively versus 0.001 g/L. The improvement of the water-solubility of butylparaben is greater than that of triclosan due to its easier complexation into the CP-β-CD cavities (i.e., butylparaben has a smaller molecular volume than triclosan). In addition, the authors reported some 2D gCOSY NMR experiments which proved the formation of inclusion complexes. The deposition of the polymer onto cellulose and the antimicrobial activity against *E. coli* and *Salmonella* revealed that triclosan was more effective. Atomic force microscopy was used to understand the biocidal mechanism of the complexes by observing the morphology of *E. coli*. When butylparaben or triclosan were used as biocidal agent in the presence of CP-β-CD, the cell membrane was not affected by the complex suggesting that the biocide/CP-β-CD complexes inhibit only the metabolism. Indeed, triclosan exhibits its antimicrobial activity by inhibition of the lipid synthesis and some enzymes whereas butylparaben is assumed to influence the mitochondrial depolarization depletion of cellular adenosine triphosphate. However, the biocidal activity of the CP-β-CD alone is clearly different from the other cationic biocides or polymers which tend to cause cell membrane lysis (see below the amphiphilic biocides section). The authors concluded that the cationic groups on the CP-β-CD facilitate the biocides approach onto the negatively charged cell membrane improving accordingly the antimicrobial activity of the biocides.

**Figure 8:**
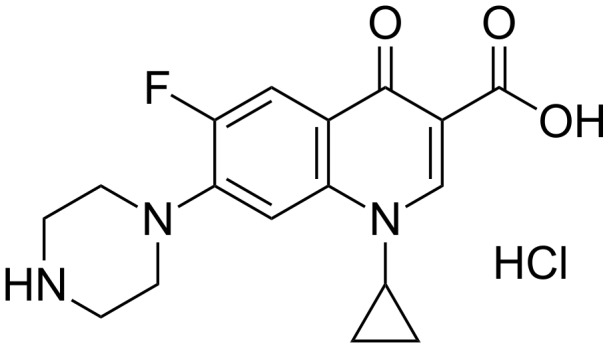
Chemical structure of ciprofloxacin hydrochloride.

In 2014, Dong et al. published on the inclusion of ciprofloxacin by grafting β-CD on cellulose fibers (Figure 8) [65].

The β-CD-grafted cellulose was prepared by the formation of CA-β-CD obtained after the dehydration of the two adjacent carboxyl groups of the citric acid (CA) to form a cyclic anhydride, which reacts with the hydroxyl groups of β-CD. Next, the CA-β-CDs were covalently bound to the hydroxyl groups of cellulose after the formation of a new cyclic anhydride. Under the best conditions ([CA-β-CD] = 300 g/L, pH 3.4, 15 min, 160 °C), the grafted ratio of β-CD onto cellulose fibers was 9.7%. The ciprofloxacin hydrochloride was encapsulated and its release behavior from cellulose fibers was also studied. The results clearly proved that the release of the biocide from the grafted cellulose fibers was prolonged. Indeed, the cumulative release from the virgin fibers was 90% within the first 30 min, while the modified ones reached the same level after 240 min because of the formation of inclusion complexes. However, the presence of β-CD and biocide increased the disorder of cellulose microstructure and modified its mechanical properties. The bacterial activity against *E. coli* and *S. aureus* exhibited excellent bacterial activity for grafted fibers loading ciprofloxacin compared to virgin ones. Indeed, the virgin fibers loading biocide were active for 4 days whereas the grafted fibers were active for 15 days.

### Amphiphilic biocides

Unlike organic biocides which have very different modes of action, the amphiphilic biocides mechanism is based on four-steps: (a) the biocide diffuses in the solution, (b) electrostatic interactions maintained the charged biocides in the vicinity of the cell membrane, (c) the biocide is inserted between the phospholipids of the cell membrane, and (d) the modification of the membrane charge induces the cell lysis. This mechanism is common for all ionic amphiphilic compounds and is well documented in the literature [66–67]. It is worth mentioning that the nonionic surfactants also act on membranes but their interaction mechanism is unknown. Some authors have proposed an interaction of the nonionic surfactants with lipid membranes by formation of channels through the membrane [68]. More recently, Groot and Rabone claimed that the inclusion of nonionic surfactants reduces the extensibility and the maximum stress that the bilayer can withstand [69]. These modifications bring some explanations for the cells lysis. Due to their structures, these amphiphilic biocides are more water-soluble than organic preservatives. Therefore, their encapsulation by CDs is generally not used to enhance their water-solubility.

#### i) Antagonistic effect

**Figure 9:**
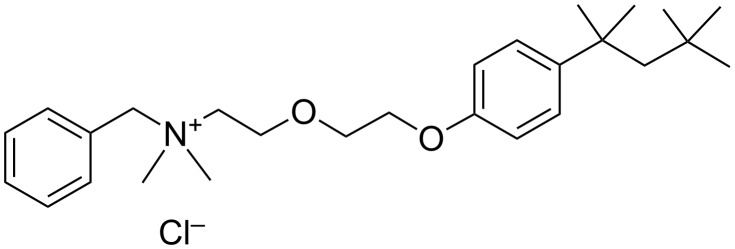
Chemical structure of benzethonium chloride.

In 1992, Simpson reported that the formation of benzethonium–β-CD inclusion complexes further improved the water-solubility of benzethonium chloride but inactivated its biocidal activity (Figure 9) [70]. Based on this observation, Simpson proposed to use the complexation to inhibit the biocidal activity of microorganism/biocide mixtures. Classically, the inactivation of quaternary ammonium biocides is performed with mixtures of polyoxyethylene (20) sorbitan monooleate (Tween 80) and lecithin which trap the biocidal agents inside the micelles formed by the neutralization agents. In his paper, Simpson showed that the neutralization can be made selectively by choosing an appropriate CD for inactivation of one or more quaternary ammonium salts or, alternatively, two or more CDs can be used to neutralize the action of several ammonium salts.

**Figure 10:**
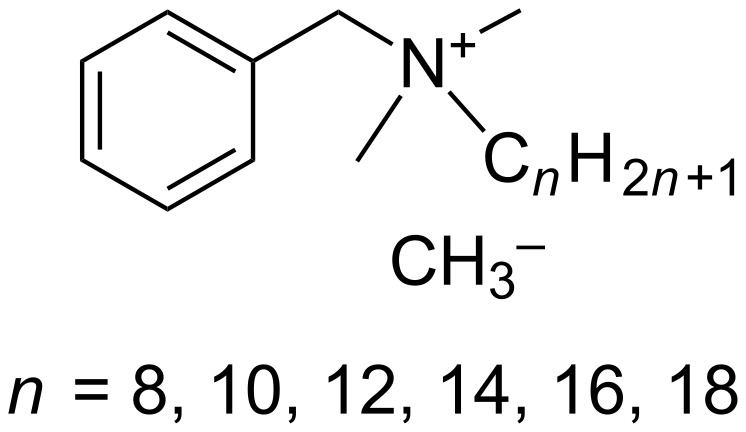
Chemical structure of benzalkonium chlorides.

The same year, Loftsson et al. reported on the interactions between benzalkonium chloride (and other organic preservatives such as chlorhexidine gluconate, chlorobutanol, methylparaben and propylparaben) and HP-β-CD (Figure 10) [71].

The authors showed that biocides could displace drug molecules (e.g., hydrocortisone, prednisolone and triamcinolone acetonide) from the CD cavity to the solution, thus, reducing the solubilizing effects of the drugs through complexation. Moreover, the antimicrobial activity of the preservatives was reduced thanks to the inclusion complexes. However, as previously mentioned for organic biocides, the effectiveness of hydrophobic preservatives was affected by the CDs. Indeed, the degree of complexation is a key parameter to obtain biocidal activity as only free preservative molecules are effective against microorganisms (see above). As a consequence, the appropriate preservative must be chosen in the case of complicated drug formulations. It is noteworthy that the use of high concentrations of preservatives may have negative biological effects for patients. These results are very similar to the results of Lehner et al. with organic biocides (see above). However, in this case, the CD effects on the antimicrobial activity of preservatives have been studied without drug molecules.

**Scheme 4:**
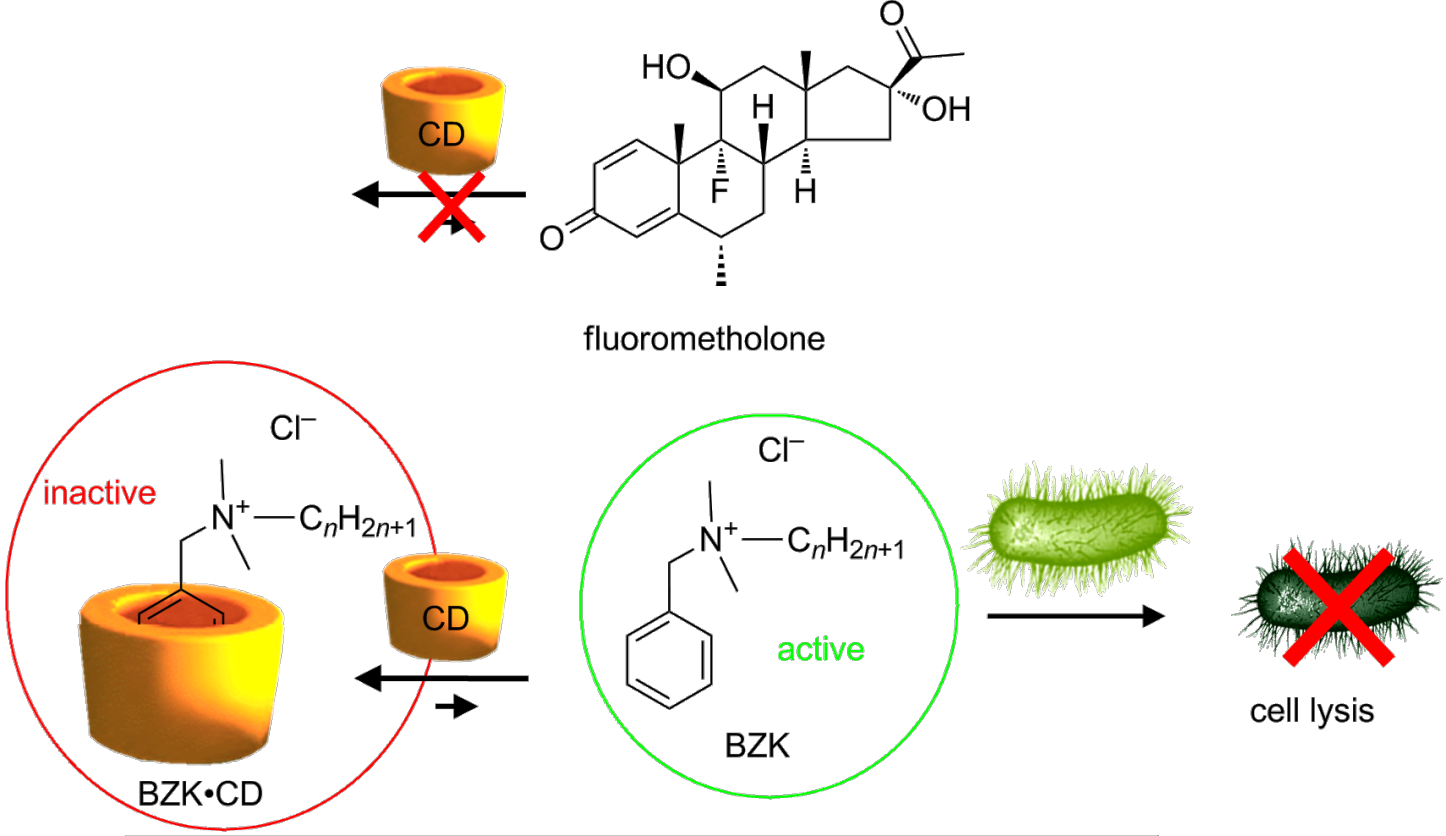
Multiple equilibria of CD with benzalkonium chloride (BZK) and fluorometholone.

To solve this drawback, Malaekeh-Nikouei et al. studied the interaction between benzalkonium chloride with HP-γ-CD and SBE-β-CD with or without ethylenediaminetetraacetic acid (EDTA, preservative potentiator) and fluorometholone [72]. Their biocidal activity against *E. coli*, *P. aeruginosa*, *S. aureus*, *C. albicans* and *Aspergillus niger* was followed over 28 days. From the results, it was clear that HP-γ- and SBE-β-CD decreased the effectiveness of benzalkonium chloride in the presence or absence of EDTA. This effect was clearly intensified when CD concentration was increased because of the formation of inactive inclusion complexes (Scheme 4). The only exception for fluorometholone solution was obtained for the following formulation HP-γ-CD 5% with benzalkonium chloride 0.02% and EDTA 0.1%. This solution was effective against tested microorganisms both in the presence and absence of drug molecules. Unfortunately, no explanation was provided by the authors. However, the complexation of fluorometholone is less easy than the complexation of benzalkonium chloride into HP-γ-CD cavities because the smaller size of benzalkonium facilitates its entering into the cavities of HP-γ-CD (see Scheme 4).

As presented in this section, the biocidal activity of amphiphilic agents can be reduced upon complexation. The mechanism is very similar to that of organic biocides (see above). The use of CDs can notwithstanding is useful to avoid co-micellization in complex formulations, by, in a certain way, immobilizing the biocide. All these points are developed in the remainder of this paragraph.

#### ii) Avoiding co-micellization of biocidal alkyl ammonium and detergent polyoxyethylated fatty alcohol surfactants

**Scheme 5:**
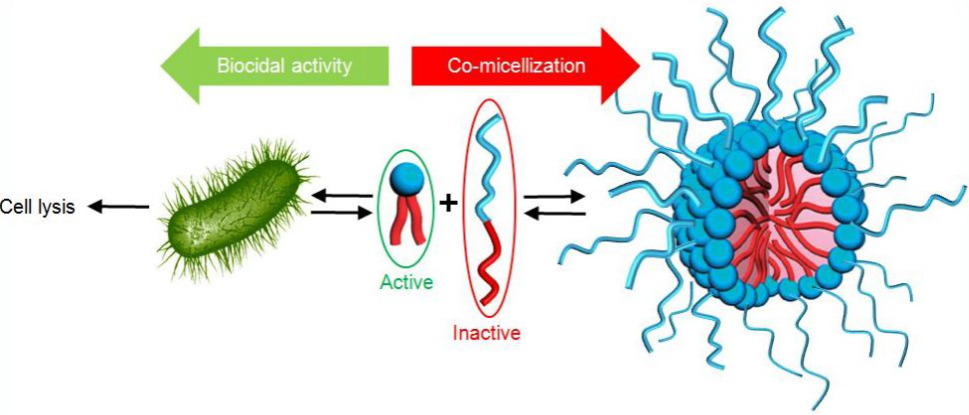
Competition between co-micellization and biocidal activity observed for didecyldimethylammonium chloride and polyethoxylated fatty alcohol binary mixtures.

Didecyldimethylammonium chloride is one of the most widely used biocides for cleaning chirurgical equipments. However, this cationic biocide is generally not used alone. Typically, the formulations also contain polyethoxylated fatty alcohols leading thus to disinfectant/detergent compositions. In such surfactant mixtures, the presence of the various amphiphiles results in a spontaneous co-aggregation obtained at lower concentrations compared to the cationic biocide alone. As the free active alkylammonium is reduced, the biocidal activity is inhibited (Scheme 5) [17]. To avoid this behavior, CDs can be used to form highly water-soluble complexes.

In this context, the encapsulation of didecyldimethylammonium chloride with various CDs (α-, β-, γ-, HP-α-, HP-β- and CM-β-CD) was investigated by Leclercq et al. [73]. The binding constants and stoichiometries were determined by combining the use of ammonium and chloride selective electrodes, NMR spectroscopy and molecular modeling. The biocidal activity of the inclusion complexes against *P. aeruginosa* was examined. All these data are reported in Table 4. As previously mentioned, the antagonistic effect is clearly shown in Table 4. However, the nature of CDs has a strongly influence on the stability and the geometries of the inclusion complexes and a relationship is obtained between these last and the biocidal activity. Indeed, as it can be deduced from the data reported in Table 4, the binding constants, the stoichiometries and the geometries are the key parameters to maintain an acceptable antimicrobial activity in the case of CD/biocide mixtures.

**Table 4:** Binding constants and geometries of various CD•didecyldimethylammonium chloride inclusion complexes and minimal biocidal concentrations (MBC) for free and complexed didecyldimethylammonium chloride against *P. aeruginosa*.^a^

CD	*K*_1_	*K*_2_	Geometries	MBC (µM)

–	–	–	–	55
α-CD	26 000	7 500	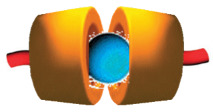	420
β-CD	9 700	2 900	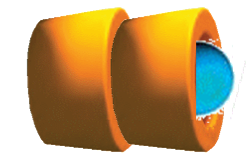	190
γ-CD	7 600	–	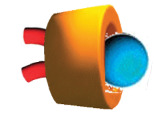	55
HP-α-CD	8 400	2 800	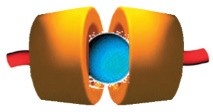	480
HP-β-CD	26 100	–	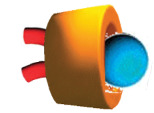	180
CM-β-CD	86 400	–	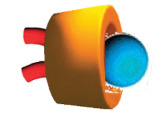	360

^a^Taken from [73].

**Scheme 6:**
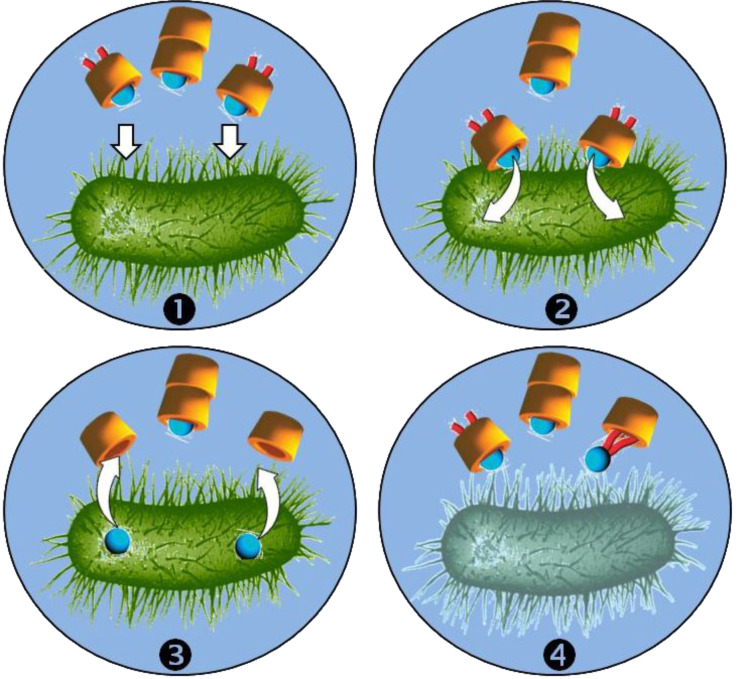
Proposed antimicrobial mechanism of encapsulated didecyldimethylammonium chloride by CDs: (1) diffusion of complexes, (2) electrostatic interaction with the cell membrane if 1:1 complex, (3) free ammonium insertion, and (4) cell lysis.

As an example, the most active complex is obtained with the γ-CD that presents the weakest binding constant and only a 1:1 stoichiometry. In contrast, the highest MBC is obtained with α-, HP-α- and CM-β-CD. For the first two, the prevalence of 2:1 complex implies a total encapsulation of the alkylammonium, i.e., the positively charge is screened. For CM-β-CD, despite the formation of a 1:1 geometry, the charge is screened by the carboxylate residues. For these CDs, the authors supposed that the target recognition is disturbed. Indeed, since the membrane of microorganisms is negatively charged, the combination of strong binding constant(s) and the charge screening is not favorable to the binding of the complex on the microorganisms. The partial biocidal activity observed for β- and HP-β-CD is only due to the high binding constant because of the absence of charge screening. Based on these results, the authors proposed the mechanism described in Scheme 6.

In 2012, the same group reported on the antifungal activity of CD–didecyldimethylammonium chloride inclusion complexes against *C. albicans* [74]. In this work, the biocidal mechanism was confirmed in vitro by monitoring the mean colony count data, the uncomplexed didecyldimethylammonium concentration and the ζ-potential over time of aqueous suspensions of *C. albicans* with or without CDs. The decrease of the free didecyldimethylammonium concentration and the modification of the ζ-potential of the microorganism are related to the insertion of alkylammonium cations in the cell membrane which disturbs the membrane potential and leads to cell lysis. Moreover, the authors also pointed out the unusual behavior of the β-CD–didecyldimethylammonium inclusion complex for which the minimal inhibitory concentrations (MIC) are weaker than those of the free alkylammonium cation. Moreover, the MIC values for α- and γ-CD inclusion complexes are similar to that of the free alkylammonium. This synergistic effect with respect to the growth of *C. albicans* can be related to the interaction of CDs and phospholipids (see below) [75–76].

**Scheme 7:**
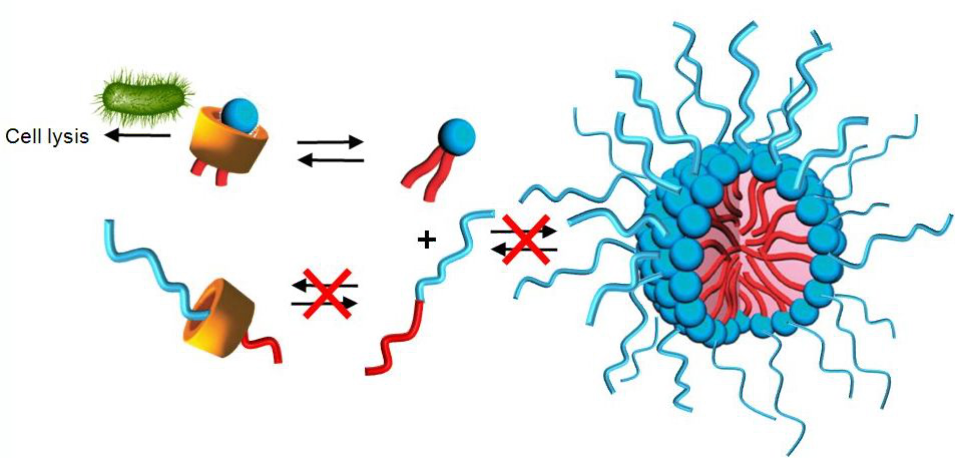
Inhibition of co-micellization process observed for didecyldimethylammonium chloride, octaethyleneglycol monododecyl ether and γ-CD ternary mixtures.

In 2013, the ternary mixtures of didecyldimethylammonium chloride, octaethyleneglycol monododecyl ether (detergent) and CDs were fully investigated from a physicochemical point of view [77]. The determination of the association constants revealed that γ-CD can be very useful to modulate the negative effect of the co-micellization. Indeed, γ-CDs only complex the alkylammonium and co-micelles do not form (Scheme 7). Moreover, this complex is also more active than the free ammonium cation against *P. aeruginosa* and *C. albicans* [73–74].

#### iii) Immobilization

**Scheme 8:**
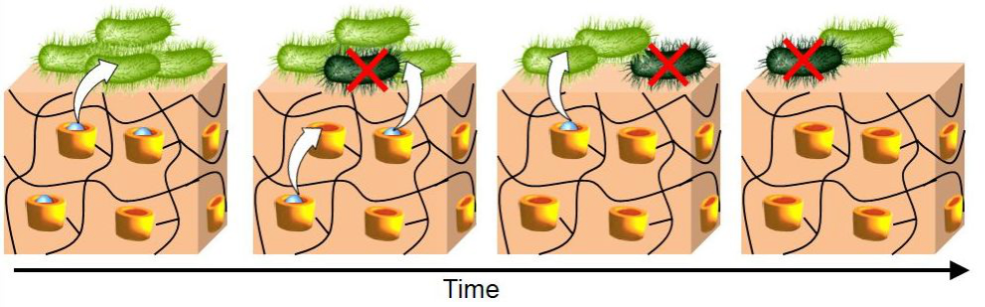
Schematic representation of biocide release from a chemically cross-linked CD network.

In contrast to the single biocide/CD complexes in solution that show a dissolution-controlled biocide release rate, delivery systems based on immobilized CDs provide diffusion- or affinity-controlled release kinetics. In this mechanism, the guest biocide released from one CD may become available to form new complexes with other available CDs during the diffusion through the cross-linking matrix (Scheme 8). The diffusion and the affinity of biocide for CDs allow to obtained unique sustained release systems with switchable properties [78].

In the context of prevention and management of wound infections, Garcia-Fernandez et al. highlighted the role of HP-β-CDs as main components of hydrogels and gauzes for the efficient delivery of antiseptics in several hours [79]. The benzalkonium chloride was chosen because of its skin antiseptic properties and its ability to form inclusion complexes with various CDs (see above). The authors have grafted CDs to cotton gauzes using citric acid as the linker. The optimal conditions were obtained at 190 °C for 15 min, leading to a grafting yield of about 148%. It is worth mentioning that the grafting yield was calculated from the difference between the final and the initial weights of the gauzes (i.e., the grafting yield represents the increase of weight of the gauzes). With the optimal conditions, the gauze was enabled to host up to 80 mg of biocide by gram of gauze. The biocidal activity against *S. epidermidis* and *E. coli* revealed that the benzalkonium chloride loaded networks inhibited the growth and reduced the number of living cells of the two strains on agar plates and in liquid medium. The authors prove that the cross-linked CDs regulate the release through an affinity-driven mechanism (see above). Moreover, the antiseptic-loaded gauzes were able to inhibit biofilm formation of *S. aureus* when applied in early stages of biofilm formation.

### Heavy-metal biocides

#### i) Silver nanoparticles

As a consequence of evolution and as a response to pressures imposed, resistance of any organism to biocides increases. In this context, the use of silver and silver-based compounds as alternative biocidal agents has emerged in the last decade. Silver nanoparticles are very attractive because of their high thermal stability, low toxicity to human cells and effective broad-spectrum antibacterial activity. The antibacterial activity of these nanoparticles is probably due to their rapid breakdown which releases ionic silver that interact with the thiol residues of bacterial enzymes [80]. As a consequence, the bacterial DNA replication is inhibited. Moreover, the bacterial cytoplasmic membranes can also be damaged leading to cell lysis [81]. In contrast to solutions of silver ions, the biocidal efficacy of the silver nanoparticles is improved because of a high specific surface-to-volume ratio leading to an increase of their contact with microorganisms, promoting the dissolution of silver ions. Their stabilization in aqueous solution is achieved using capped agents (polymers, sodium dodecylsulfate, etc.). Since 2004, the use of β-CD as a silver nanoparticle stabilizer has been described in several papers [82–83].

**Scheme 9:**
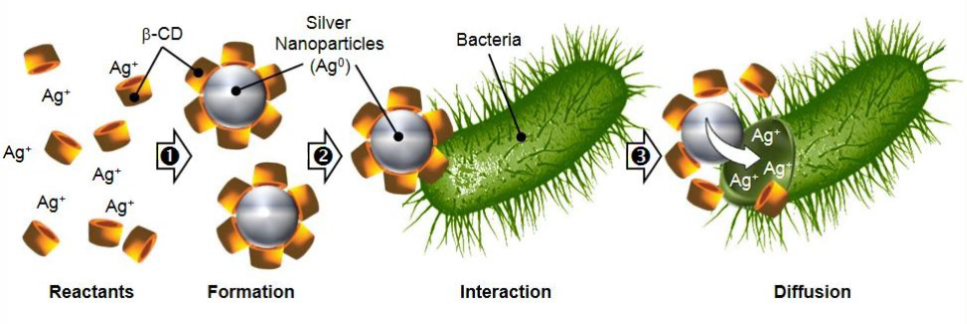
Proposed Trojan horse mechanism of silver nanoparticles capped by β-CD.

In 2010, Jaiswal et al. proposed to use of β-CD as a capping agent [84]. The reduction of silver salts using sodium borohydride followed by β-CD capping allows the synthesis of stable spherical silver nanoparticles. The authors proved by dynamic light scattering that the average diameter of the capped particles was smaller than uncapped ones (up to 3-fold). Moreover, these nanoparticles have superior photostability to intense ultraviolet radiation and significantly higher antibacterial activity (up to 3.5-fold) were obtained against *E. coli*, *P. aeruginosa* and *S. aureus*. As the β-CD concentration delay bacterial growth, the authors proposed a Trojan horse mechanism enhancing silver ion absorption inside the bacterial cell (Scheme 9).

In 2010, George et al. reduced silver acetate with dodecylamine to obtain silver nanoparticles which were subsequently transferred from the organic to the aqueous phase containing β-CD as a capping agent [85]. The “β-CD/silver” nanoparticles were purified and characterized by spectroscopic and microscopic analyses. The results showed that the encapsulation confered a considerable stability to the nanoparticles. Moreover, the silver nanoparticles were efficient in vitro against *P. aeruginosa*, *S. aureus*, *Serratia marcescens*, *E. coli* and *Klebsiella pneumoniae*. In 2011, the same group extended the scope of microorganisms to human opportunistic pathogenic fungi such as *Aspergillus fumigatus*, *Mucor ramosissimus* and *Chrysosporium* species [86]. Similarly, Priya et al. used sodium citrate as a reducing agent followed by capping with β-cyclodextrin for the preparation of silver nanoparticles [87] with an increased biocidal activity against *P. aeruginosa* and *S. aureus* of the β-CD capped silver nanoparticles compared to the uncapped ones. The proposed mechanism is the same as that of Jaiswal et al. (see Scheme 9) [84].

In 2010, Bajpai et al. loaded silver ions (Ag^+^) on CD-grafted onto cotton fabric to achieve an antimicrobial property [88]. A correct antibacterial activity was obtained against *E. coli* and the release of silver ions was observed for a period of seven days. Popescu et al. also reported in 2013 the antibacterial action of silver applied on cellulose fibers grafted with MCT-β-CD [89]. Through a completely ecological process, without using dispersants, the authors could “link” the silver nanoparticles (Ag^0^) or silver ions (Ag^+^) in situ on cotton via the cellulose functionalized by CDs. FTIR spectra revealed that the stabilization occurred by the interactions between the hydroxyl groups of the grafted CD and the silver ions or the nanoparticles. The binding constant of the complexes formed between Ag^+^ and CD was determined by potentiometric titration whereas the stability constant of the Ag^0^–CD complex was determined from the UV–vis spectra. The constants were estimated at 360 and 3.07 × 10^6^ M^−1^ for Ag^0^ and Ag^+^, respectively. Antibacterial activities against *E. coli* and *S. aureus* were determined for the fabrics grafted with CD and treated with Ag^0^ or Ag^+^. The antibacterial activities were very similar for the two fibers (i.e., CD/Ag^0^ and CD/Ag^+^) suggesting that the nanoparticles were dissociated into Ag^+^, i.e., the active species. Moreover, the antibacterial effect of grafted fabrics, washed 10 times, was equal to the unwashed original fabric. Finally, an interesting relationship was established between the stability constants of the CD/Ag^0^ and CD/Ag^+^ complexes and the antibacterial activity as well as the resistance to washing.

In 2014, Andrate et al. reported on the synthesis of silver nanoparticles from an aqueous silver nitrate solution with glucose as a reducing agent, sodium hydroxide as a reaction catalyst and β-CD as a stabilizer [90]. They also investigated the structure and morphology of the stabilizing layer around the silver nanoparticles. The modification of the surface of the nanoparticles by β-CD thus demonstrating the interaction between the hydroxyl groups of the β-CD and the nanoparticles’ surface was confirmed by Raman spectroscopy. Transmission electron microscopy images showed pseudo-spherical nanoparticles (Ø ≈ 28.0 nm). The authors characterized the β-CD layer surrounding the nanoparticles by a novel complementary analytical electron microscopy based on electron spectroscopy imaging in the transmission microscope. The existence of a uniform and interacting β-CD layer covering the nanoparticles (i.e., the presence of carbon and oxygen atoms was detected in the surface) was unambiguously proved. Finally, the antibacterial activity against *E. coli*, *P. aeruginosa* and *S. aureus* strains was investigated. The results were very promising especially against the *E. coli*. Indeed, the minimum inhibitory concentration of silver nanoparticles for a 2 h exposure to the *E. coli* microorganism was 20 μg/mL.

#### ii) Other metallic nanoparticles

Various nanoparticles have been successfully developed as antimicrobial agents. As an example, zinc oxide and titanium dioxide nanoparticles have been prepared by chemical and sol–gel methods [91]. Biocidal applications of these nanoparticles may lead to valuable discoveries in various fields such as medical devices and antimicrobial systems [92].

**Scheme 10:**
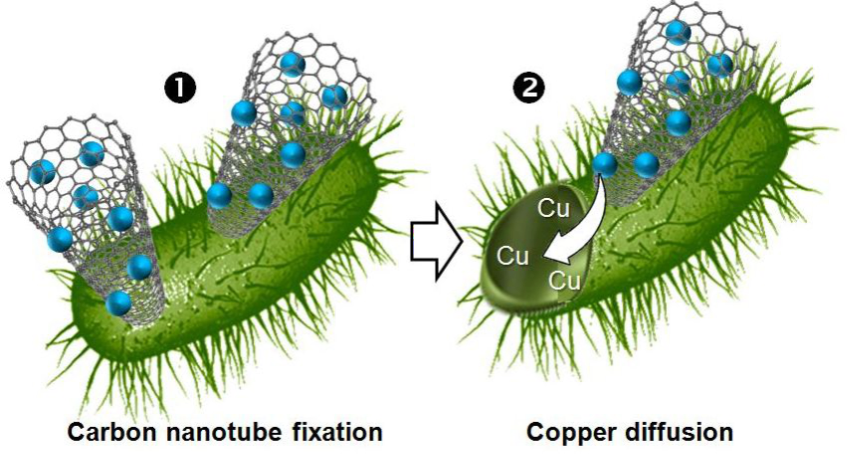
Proposed mechanism of copper nanoparticles immobilized on carbon nanotube and embedded in water-insoluble cyclodextrin polyurethane polymers.

In this context, Lukhele et al. explored the use of copper nanoparticles impregnated on CD polyurethanes for possible use in water disinfection [93]. Carbon nanotubes embedded in water-insoluble CD polyurethane polymers are used to immobilize copper and silver nanoparticles. The resulting material was fully characterized and the antibacterial properties using spiked water samples containing *E. coli* and *Salmonella typhi* were evaluated in the presence of *p*-nitrophenol (organic pollutant). The material based on polyurethanes reduced up to 3 logs of bacteria and adsorbed up to 55% of *p*-nitrophenol pollutant. It is worth mentioning that single-walled carbon nanotubes already exhibit a strong antimicrobial activity because of the piercing of the bacterial cell membranes (Scheme 10) [94]. Therefore, their combination with biocidal nanoparticles allows a synergism behavior: the carbon nanotubes promote the nanoparticles penetration into the cytoplasm by piercing of the bacterial cells. Indeed, the combined action of carbon nanotubes and biocidal nanoparticles is higher than the sum of the activities of any actives on their own.

Based on this ascertainment, the following antimicrobial mechanism was proposed: i) the carbon nanotube embedded in water-insoluble cyclodextrin polyurethane polymers pierce the bacterial cell membrane by direct contacts, and ii) the copper nanoparticles release copper inside the bacterial cell. The combination of these two steps induces bacterium autolysis [95].

In 2012, Selvam et al. proposed to use S-β-CD crosslinked cotton fabric to improve the biocidal activity of ZnO, TiO_2_, and Ag nanoparticles coating [96]. Ethylenediaminetetraacetic acid was used to crosslink the cyclodextrin with cotton fabric. The three nanoparticles were coated on crosslinked cotton fabric. The biocide efficiency against *S. aureus* and *E. coli* decreases in the following order: ZnO > Ag> TiO_2_. Moreover, the authors mentioned that the S-β-CD crosslinked ZnO nanoparticles coated fabric have the lowest production costs. This type of cellulose fabric may be suitable for medicinal bandages, etc.

In 2014, Li et al. synthesized β-CDs/polyacrylonitrile/copper nanorods composite fibers [97]. The synthesis included two steps: i) the preparation of the β-CDs/polyacrylonitrile composite fibers by electrospining, and ii) the preparation of the β-CDs/polyacrylonitrile/copper nanorods composite fibers by adsorption and reduction. The composite fibers were fully characterized by FTIR, SEM, TEM, energy dispersive spectroscopy and X-ray photoelectron spectroscopy. The results indicated that the copper nanorods were not only successfully synthesized on the surface of the composite fibers but also the copper nanorods were distributed without aggregation on the composite fibers. The antibacterial efficacy of the composite fibers against *E. coli* indicated that they have bactericidal effects only in the presence of copper nanorods. Based on the literature, the authors mentioned than the loaded copper ions interact with bacterial cell membranes which induced structural change and leading to cell death. The proposed system can be useful to water purification systems and filtration membrane.

### Intrinsic biocidal activity of CDs and synergism effect

As mentioned above, CDs have mainly been used as excipients which increase the solubility and the stability of biocides, mask their unpleasant odor, etc. (see above). Nevertheless, a large number of studies suggests that native and modified CDs can be used as active biocides in the treatment and prevention of several host–pathogen infections. The biological effects of the CDs can be classified according to their role: i) as complexing agents of endogenous substances, ii) as complexing agents of exogenous substances and iii) as blockers endogenous and exogenous macromolecules [98]. In this review, only the complexation of endogenous substances (i.e., lipids, cholesterol) is considered.

#### i) Impact of CDs on the cell membranes

The hemolytic activity of native CDs is well known and follows the order: β-CD > α-CD > γ-CD [99]. The differences between these CDs are ascribed to their cavity size. Indeed, CDs are able to solubilize and to extract some phospholipids (e.g., phosphatidylcholine and sphingomyelin) from the cell membrane [100]. Moreover, the solubilization/extraction process is not limited to phospholipids. Indeed, the cholesterol extraction from the red blood cell membranes results in an increase of the membrane fluidity leading to the cell lysis [101]. It is noteworthy that CDs also remove proteins from red blood cell membranes [102].

#### ii) CDs as virucides

In the literature, some authors have reported that native or modified β-CDs alone can be used as virucides. This virucidal activity has been demonstrated for the poliovirus [103], the human T cell leukemia virus (HTLV-1) [104], the varicella-zoster [105], the hepatitis B virus [106], the HIV-1 [107], etc. The precise antiviral mechanism is still unknown but the antiviral activity might be due to lipids, proteins and cholesterol extraction from the cell membranes (see above). As an example, Leydet et al. investigated the antiviral activity of several charged CD derivatives and demonstrated anti-HIV and anticytomegalovirus activity, but found no anti-HSV activity [108]. On the other hand, Liao et al. reported that HP-β-CD has an in vitro activity against HIV. Indeed, the virus inactivation was dose-dependent and the activity was likely attributable to loss of cholesterol [109]. Since the cholesterol in HIV is strictly required for fusion and infectivity, HP-β-CD is an excellent candidate for its use as a chemical barrier for AIDS prophylaxis.

In 2003, Wallace et al. reported that the native β-CD provides almost complete protection to Vero cells against the cytopathic effect caused by HSV-1 or HSV-2 infections [110]. The authors indicated that β-CD reduced both cell-free and cell-associated viruses more effectively than acyclovir (the most commonly used antiviral drug for the treatment of HSV infections). Moreover, β-CD had marked antiherpetic activity against acyclovir-resistant strain of HSV-1. The authors also suggested that β-CD worked at a different step of the virus replication, which could have a major clinical significance. Moreover, suboptimal concentrations of β-CD in combination with acyclovir showed an additive protective effect when Vero cells were infected. The CD would exert its mechanism of action at an early stage of the viral replication. Moreover, this antiviral activity was clearly dependent on the cavity size. Indeed, α-CD exhibited no significant antiviral activity against HSV-1 and HSV-2 in infected Vero cells at final concentrations up to 8 mg/mL, while, under the same conditions, β-CD showed significant anti-HSV-1 and HSV-2 activity even at very high multiplicity of infection.

#### iii) Extension to other pathogens agents

Since lipid and cholesterol extraction is general but dependent on the membrane structure, a whole range of studies have shown reduction or inhibition of entry of different infections in host cells due to the presence of CDs. This effect has been demonstrated for several bacteria, fungi and parasites: *Plasmodium* species [111], *Campylobacter jejuni* [112], *Leishmania donovani* [113], *Bacillus* [114], etc.

#### iv) Towards a synergistic effect?

Since the CDs are able to solubilize lipids, cholesterol and proteins from the cell membrane and since their biocidal activity is obtained at relatively high concentrations, it is likely that their combination with biocides (active at weak concentrations) results in a synergism in terms of biocidal activity. Similar conclusions have been drawn by Boldescu et al. from the data available in the literature [115–116]. Indeed, the authors proposed that the cholesterol complexation by CDs increases the permeability of the mycobacterial cell wall and promotes penetration of antimycobacterial compounds inside. This assertion is supported by the work of Brzostek et al. that demonstrated the cholesterol extraction from the *Mycobacterium tuberculosis* cell wall in the presence of CDs [117]. As a consequence, the protective lipid is disorganized and the permeability of biocides is increased. Moreover, Boldescu et al. reported the first results of biological tests performed with β- and SBE-β-CD associated with an oxadiazole derivative. Their results revealed that the antituberculosis activity is only improved with the native CDs and not with the modified one. The inefficiency of the SBE-β-CD is attributed to its anionic nature which limits the cholesterol complexation.

## Conclusion

**Scheme 11:**
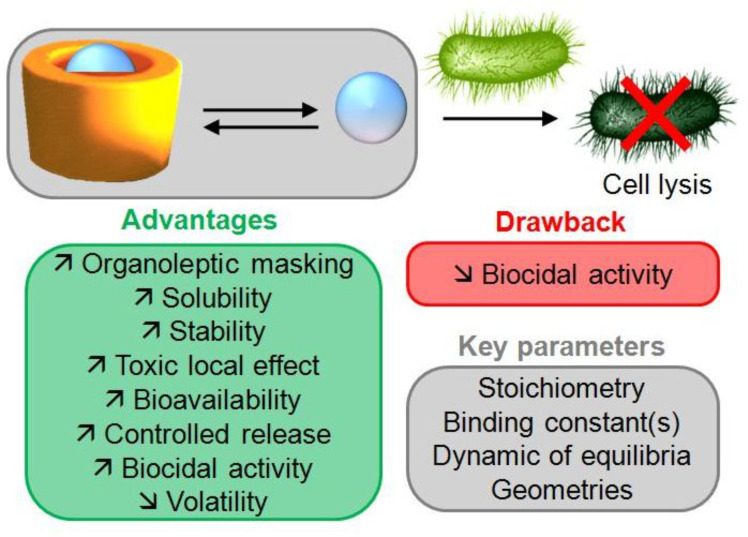
Advantages and drawback of the physicochemical and biopharmaceutical properties of CDs/biocides inclusion complexes, and key parameters to control the biocidal activity.

This review presents an analysis of current and potential applications of CDs for the encapsulation of biocides. All applications are based on the use of CDs as complexing agents. The formation of inclusion complexes with biocides presents several advantages (see Scheme 11).

As depicted in Scheme 11, the formation of inclusion complexes is able to increase or decrease the biocidal activity. This behavior comes from a balance between microscopic and macroscopic effects. Indeed, on the microscopic scale, inclusion of relatively soluble biocides induces a reduction of free biocides (i.e., the active species), which might reduce the biocidal activity (i.e., the minimal inhibition or biocidal concentrations increase). On the macroscopic scale, complexation of slightly soluble biocides improves their apparent solubility, which can increase the available quantity of biocide and thus enhance the biocidal activity. Accordingly, if the biological study is carried out with a biocide concentration inferior or close to the intrinsic biocide solubility (for both the biocide alone and the complex), the presence of CDs is expected to lower the observed biocidal activity. On the contrary, if the biological study takes profit of the increased solubility of the complex, an increase of the biocidal activity can be observed. On the other hand, the controlled release is probably one of the most important advantages. Indeed, an inherent and essential property of supramolecular systems is their reversibility. For most of the CD complexes, the complexation kinetics is extremely fast. Accordingly, the delayed release which is generally observed in the presence of CDs is not linked to kinetics of inclusion but is rather induced by the thermodynamic control. Indeed, at a given time, only a certain percentage of biocide is free and really bioavailable (even if each encapsulated biocide might dissociate from the complex). Thus, the prolonged release comes from displacement of the inclusion equilibrium. Therefore, the equilibrium involved in the complexation and the competition with biological processes must be rationalized. This rationalization combined with the biocidal activity of the CDs alone must allow synergistic effects that become the new Holy Grail in the coming years. Moreover, the encapsulation of amphiphilic biocides and the stabilization of biocidal nanoparticles are also very promising to prepare new biocidal formulations.
